# Supplementary material for: Are carbon emissions trading and green financial instruments synergistic? -Comprehensive quantitative research based on content analysis
Source: PLoS One. 2024 Mar 7;19(3):e0298601. doi: 10.1371/journal.pone.0298601 (PMC10919601; doi:10.1371/journal.pone.0298601)
Supplement: S1 Data — (ZIP) [file pone.0298601.s001.zip › Data/地级市Z_F工作报告.zip!/║■─╧/╧μ╠╢╩╨/└┤╫╘╣1⁄2╓┌║┼í╛┬φ┐╦╩2╛▌═°í┐_2016.docx]

各位代表:

　　现在,我代表市人民政府向大会作政府工作报告,请各位代表连同《湘潭市国民经济和社会发展第十三个五年规划纲要(草案)》一并审查,并请各位政协委员和其他列席人员提出意见。

　　一、“十二五”和2015年工作回顾

　　过去五年,在省委、省政府和市委的坚强领导下,在市人大、市政协的监督支持下,我们紧密团结和依靠全市人民,积极应对经济下行压力,统筹稳增长、调结构、促改革、惠民生、防风险,创造性开展工作,总体完成了“十二五”目标任务。

　　——综合实力显著提升。地区生产总值由2010年的894亿元增加到1703.1亿元,五年年均增长11.6%;人均GDP突破6万元,居全省第二。规模工业增加值达到876.1亿元,年均增长12.9%;财政总收入达到173.9亿元,年均增长18.1%;社会消费品零售总额达到520.6亿元,年均增长14.7%。五年累计完成固定资产投资6068亿元,是“十一五”的3.2倍。存贷款余额分别达1750亿元、1295亿元,累计新增贷款723亿元,年均增长18.4%。五年到位外资35亿美元,完成进出口总额116亿美元。湘潭经开区晋升为国家级经开区。湘潭综保区获批。湘潭获得国家自主创新示范区、全国红色旅游融合发展示范区、国家现代农业示范区等20项国家级品牌。

　　——经济结构不断优化。三次产业结构由10.7:55.9:33.4调整为8.3:54.8:36.9。高耗能行业占比由50%以上降至30%以下,高加工度工业占比由39.4%升至54%以上,高新技术产业增加值占GDP比重由22.6%升至31.5%,中国驰名商标由11件增加到35件。五年共有97项科技成果获国、省科技进步奖,363吨级电动轮自卸车、“海牛”深海钻机等一批技术跻身世界一流。产业结构由过去“一钢独大”,蝶变为先进装备制造成为第一大产业,汽车及零部件、新能源等6大产业加速崛起的新格局。五年新增规模工业企业191家。吉利汽车、泰富重装、湘电集团成长为百亿企业。服务业对经济增长的贡献率由29.7%提升到49.3%。步步高集团成长为湖南零售冠军企业。规模以上农产品加工企业增加值达150亿元,是2010年的3倍多。非公经济占比由52%升至67%。园区规模工业增加值占比由41.2%升至71.3%。

　　——城乡面貌焕然一新。河东中心城区功能大幅完善,河西旧城改造有序推进,九华新城初具规模,昭岳、天易等新城片区稳健崛起。基础设施进一步配套完善,通车高速公路由4条增加到7条,新(改、扩)建城市主次干道75条,完成湘江风光带建设19.2公里,新建干线公路27条、630余公里,硬化农村公路3030公里,构成全国地级市最密集的公路网;沪昆高铁设站湘潭和韶山,湘潭迈入“高铁时代”。城市绿荫行动有力推进,人均公园绿地面积达到9.2m2。大力创建全国文明城市,进入全国文明城市提名城市行列。城乡统筹步伐加快。城镇化率达58.6%。花石、棋梓、银田3个特色镇和梅林桥城乡统筹示范片、姜畲现代农业示范区建设推进良好。五年完成各类水利工程13.5万处,解决82万农村人口饮水安全问题。农村环境卫生综合整治持续推进,城乡面貌显著改观。

　　——“两型”建设卓有成效。实施湘江保护和治理“一号重点工程”和大气污染防治行动计划,累计关停淘汰退出涉重金属企业94家,竹埠港地区等重点区域环境综合整治在全省率先突破,湘江城区段全面禁止采砂,湘潭段水质常年稳定在Ⅲ类,城市空气质量优良率、污水处理率分别达到75%和92.5%。生态建设五年完成营造林125.3万亩,综合治理水土流失面积92.8km2,复绿矿山63处,增加湿地保护面积1.1万公顷。昭山地区保护与发展机制不断完善,成为长株潭“绿心”。全市森林覆盖率达46.2%以上。推进“两型”示范创建,韶山成为全省第一个“两型”综合示范片区。“两型”生产生活方式逐步普及。万元GDP综合能耗由1.17吨标准煤下降到0.82吨标准煤,全面完成“十二五”主要污染物减排目标。人居环境明显改善。

　　——民生保障持续改善。民生支出占公共财政支出比重提高到63%以上。五年累计新增城镇就业28万人,新增农村劳动力转移就业13万人。城乡居民人均可支配收入分别达29237元和15347元,年均增长10.9%和13.2%,城乡居民收入比由2.35∶1缩小至1.89∶1。社会保障水平保持全省前列。竣工保障性住房和棚户区改造6.4万套,农村危房改造20431户,最低收入住房困难家庭实现应保尽保。住房公积金增值收益支持廉租房建设补充资金3.4亿元。减少农村建档立卡贫困人口5.97万人。大力推进教育优质发展,五年建成合格学校352所、公办幼儿园54所。医药卫生体制改革深入推进,新农合参合率达98.28%。人口计生工作连续五年被评为全省模范。文体事业蓬勃发展,“欢乐潇湘·幸福湘潭”“全民健身挑战”等活动深受群众喜爱。大型实景剧《中国出了个毛泽东》演出439场,观众达65万人次。法治湘潭建设持续推进,社会治理不断创新,人民群众安全感和满意度不断提高,和谐稳定的社会局面进一步巩固。

　　——制度建设日臻完善。推进全面深化改革,大力创新体制机制,行政管理体制改革、国企改革、商事制度改革、投融资和财税体制改革、社会领域改革等取得重大突破,发展活力进一步释放。以制度建设规范行政行为,修订完善《市政府工作规则》,健全重大行政决策机制,加强行政决策合法性审查,完善政府投融资公司“三重一大”决策制度。规范完善国土、规划管理制度,成立市国土资源管理委员会和城乡规划委员会,加强对国土和规划的集中规范管理。制定实施全市统一的征拆政策,严肃查处政府投资建设项目工程造价计价不良行为,对市本级政府投资项目全面推行预算、结算、决算“三审制”。推进服务型政府建设,市政务服务中心、公共资源交易中心、“12345”社会求助服务平台建成运行。深入开展党的群众路线教育实践活动和“三严三实”专题教育,严厉整治“四风”,加强廉政建设,政府系统工作人员规矩意识、服务意识和行政效能不断提升。

　　各位代表,2015年是“十二五”收官之年,也是经济运行特别艰难的一年。我们坚决贯彻落实国省一系列重大决策和政策措施,奋力攻坚克难,较好地完成了市十四届人大三次会议确定的目标任务,为“十三五”顺利起航巩固了基础。全年地区生产总值同比增长9.6%,规模工业增加值增长8.2%,固定资产投资增长20.1%,财政总收入增长9.1%,社会消费品零售总额增长12.8%,城乡居民人均可支配收入分别增长8%和8.9%。

　　1.抓产业、扶企业,稳住了经济增长。落实国省市三级惠企政策,市本级设立1亿元产业扶持资金,强化政府融资性担保公司作用,开展“送服务、解难题、稳增长”专项活动,对重点企业实行联点帮扶,总体稳住了企业发展,6大重点产业完成工业增加值599.2亿元,同比增长14.4%。启动国家自主创新示范区建设。高新技术产业增加值增长18.8%。大力发展现代服务业。步步高广场二期开业,中国(中部)岳塘国际商贸城、万达广场等专业市场和大型城市综合体加快建设。电子商务快速成长,阿里巴巴湘潭产业带上线。举办中俄红色旅游合作交流活动,获批全国红色旅游国际合作创建区。国家级红色旅游服务业标准化试点通过验收。壶天镇入选第三批全国特色景观旅游名镇。全市完成服务业增加值628.5亿元,增长12.8%。国家现代农业示范区建设稳步推进,完成农业产业化总产值580亿元,增长11%。农业产业结构转变为“猪菜粮”结构。全力打好粮食收购攻坚战,全年收购粮食38万多吨,圆满完成地方粮食储备任务。

　　2.抓项目、扩投资,强化了发展支撑。全年组织实施重点项目318个,完成投资800亿元。突出攻克项目供地征拆难题,完成土地征收1560亩、房屋征拆56万m2。国土资源管理日趋完善,资源保障更加科学。实施全市统一的招商引资政策,实际利用外资9.2亿美元、内资238亿元,成功引进吉利新能源SUV基地、蓝思科技蓝宝石产业园、恒大翡翠华庭、富力地产等重大项目。积极拓展融资渠道,实现直接融资58亿元。完善重大项目推进机制,实行“四个优先”、“五级”协调、并联审批,严格开竣工管理,威胜电气产业园一期、屹丰汽车一期、珠江啤酒等一批项目如期投产。全市完成固定资产投资1805.4亿元,新兴产业投资占比达40%,提高7.3个百分点;民间投资占比达到69.6%。投资对经济增长的贡献率达58%。

　　3.抓县域、强园区,培厚了发展优势。着眼多极增长、多点支撑,全力推进县域崛起、园区强盛。湘潭县、湘乡市进入全省全面小康经济强县十强。湘潭县积极推进“一区多园”,带动乡镇工业发展提速。湘乡市加快传统产业转型升级步伐,发展基础日益夯实。韶山市积极推进核心景区建设,旅游管理和服务得到加强。雨湖区获批国家新型工业化军民结合示范基地。岳塘区金融商务等现代服务业集聚区渐成规模。湘潭高新区获批国家创新型特色园区和全省首家国家级大学生科技创业园,与深圳清华大学研究院共建长株潭创新中心。湘潭经开区获批全省首家国家“智慧城市”试点园区,成立第二家院士工作站,与上海交大合作共建汽车工程联合研发中心。昭山示范区“三纵四横”骨干路网全面贯通,学校等公共服务设施加快完善。海工装备等4个省特色产业园建设正式起航。创新创业园区“135”工程建成标准厂房106万m2,引进企业449家。园区规模工业增加值达625亿元,增长10.8%。

　　4.抓建设、重管理,改变了城乡面貌。城市总体规划修改基本完成,编制城市色彩、排水防涝等专项规划10余项。重大基础设施建设加快推进。沪昆高铁湘潭北站站前广场投入使用。长株潭城际铁路湘潭段进展顺利。九华大道、昭云路等4条城际干道和东二环、芙蓉大道湘潭县段、潭锰路等全线通车。北二环提质改造全面完成。G320湘潭绕城线三期、杨梅洲大桥开工建设。着力解决城市拥堵问题,打通宝塔南路、白马湖路等城市断头路,四大桥取消收费,城区公共自行车租赁系统投入运行。湘潭成为全国首批综合运输服务示范城市。湘江风光带九华段全面拉通。福星中路等26条主次干道完成提质改造。九华湖公园、木鱼湖公园、滴水湖公园等对外开放。全面完成“裸露山地”绿化。消除城区照明暗区盲点802处。深化“六乱”治理,推进市容环境“五项整治”,开展拆违控违大行动,拆除违法建设140万m2、户外广告17.3万m2,二环线内中心城区立柱广告实现清零。强力治理超限超载,“双超”率下降到0.3%以下。燃气安全整治取得实质成效,东湖气柜停止运营。新一轮农村环境卫生综合整治深入推进。城镇生活垃圾无害化处理率达到100%。在全省率先以政府购买服务方式实施河道保洁,湘江干流两岸1公里范围内规模养殖全部退出。全市小煤矿全部关闭到位,矿区治理成效显著。严格执行“新环保法”,在全省率先建立环保公安联动执法室,查办全国首例环保行政拘留案件和全省首例大气污染入刑案件。城市空气优良率提高4个百分点。

　　5.抓改革、扩开放,增强了发展活力。完成政府机构改革,市政府工作部门精简至32个。取消、承接、下放行政审批事项61项、36项、33项。编制市本级权力清单和责任清单,行政权力事项减少47%。全面完成事业单位分类。乡镇区划调整改革合并撤销乡镇8个。国企改制扫尾全面完成。湘钢等企业办社会职能分离移交进展顺利。“三证合一、一照一码”登记制度正式实施,全市新增市场主体1.7万余户。推进财税和投融资体制改革,专项资金全面实行绩效管理,政府性债务得到有效控制。全面推进县级公立医院改革,村卫生室基本药物制度覆盖率达98.4%,医保支付制度改革和健康教育“湘潭模式”全国推广。在全省率先完成市、县两级不动产统一登记职责整合和机构设置。国有林场改革全面完成。农村土地承包经营权确权登记颁证工作全面铺开。大力发展开放型经济,全市完成进出口总额21.5亿美元。

　　6.抓民生、保稳定,促进了社会和谐。圆满完成省、市为民办实事任务。城乡居民就业在稳定总量的基础上结构逐步优化。退休职工养老金人均增加194元/月。生育保险统筹支付标准达到全省最高水平。低保家庭、重特大疾病医疗救助政策范围内救助比例提高至70%。特困供养人员基本医疗实现“零支付”。政府购买养老服务补助标准提高到200元/人·月。改扩建养老院8所,新增养老床位1034张。“两房两棚”建设圆满完成省定任务。特困户建房补助资金提高到4万元/户。全市住房公积金贷款额度增长率达40.11%。促进教育均衡发展,建成农村公办幼儿园9所、义务教育合格学校64所,城区新增义务教育学位2803个。湘潭成为全国首批职业教育现代学徒制试点城市。提升医疗卫生服务水平,8个乡镇卫生院、161个村卫生室建成投入使用。成功举办第四届齐白石国际文化艺术节。大型花鼓戏《齐白石》获湖南艺术节田汉大奖。全民健身示范城市创建深入推进,经常性参加锻炼的人口比例达40.5%。市党史馆、博物馆、规划展示馆建成开放。实施精准扶贫,全年脱贫4.3万人,市城区和韶山市基本实现建档立卡贫困人口全部脱贫。食品药品安全保障水平稳步提高。社会治安综合治理持续加强,安全生产形势稳定向好。认真执行市人大及其常委会决议、决定,自觉接受人大、政协和社会各界监督,办理人大代表建议和政协提案859件,办结率100%。妇女儿童、青少年、老龄、民族宗教、人防、韶灌、移民、气象、水文、广播电视、档案等工作取得新进步。驻潭人民解放军、武警部队、民兵预备役部队和人民警察在抢险救灾、维护社会稳定、支持地方经济社会发展中作出了重要贡献。

　　各位代表,过去五年,经济下行压力逐年加大,在市委的坚强领导下,全市上下进行了艰苦卓绝的奋斗,成绩来之极为不易。在此,我代表市人民政府,向辛勤工作在各行各业的广大干部群众,向人大代表、政协委员,向各民主党派、工商联、人民团体、无党派人士和离退休老同志,向驻潭部队、武警官兵和政法干警,向所有关心、支持湘潭改革发展的各界人士,表示衷心的感谢!

　　面对发展新常态和人民群众新期盼,我们清醒地认识到,政府工作的差距还很大,前进道路上还有不少困难和问题。自主创新能力不强,经济发展质量和效益不高,产业结构层次偏低,转型升级任务艰巨;城市能级较小,规划、建设、管理还比较粗放,旧城区、城中村和棚户区改造任务繁重,面临形象提升和功能完善双重压力;县域经济、镇村经济等短板明显,统筹城乡发展难度较大;市场机制还不完善,开放型经济水平不高,外拓竞争实力偏弱;民生保障和公共服务还需继续完善,部分群众生活还比较困难,提前全面建成小康任务艰巨;部分干部服务意识和工作能力还不强,行政效能有待进一步提高,等等。对此,我们必须高度重视,切实加以解决。二、“十三五”和2016年工作的目标、任务和重点

　　“十三五”时期是我国全面建成小康社会的决胜阶段。全球经济处在金融危机后的深度调整期,国际产业、科技、贸易竞争加剧,新型竞合成为国际发展大趋势。我国发展仍处于可以大有作为的战略机遇期,但机遇期内涵发生深刻变化,发展进入速度变化、结构优化、动力转换的新常态,去产能、去库存、去杠杆、降成本、补短板等结构性改革提上重要日程。湖南地处“一带一部”,面临“一带一路”、长江经济带等重大战略机遇,进入区域发展黄金期。省委省政府确立以世界眼光谋划长株潭城市群发展,将其放在全省首要位置,打造长江中游城市群核心引领区,推动长株潭地区率先迈向基本现代化。湘潭物质技术基础日益夯实,引导要素集聚、率先发展的比较优势正在加强。我们必须审时度势,提振信心,实现更高水平的发展。

　　总体要求:深入贯彻落实党的十八大和十八届三中、四中、五中全会精神,按照“四个全面”战略布局,牢固确立创新、协调、绿色、开放、共享发展理念,坚持发展第一要务,以提高经济发展质量和效益为中心,以改革创新为动力,推进“三量齐升”、“五化同步”、转型发展,将湘潭建设成中部地区崛起智造谷、全国红色旅游融合发展示范区、国家现代农业示范区,在全省率先实现城乡统筹发展,率先建成两型社会示范区,率先全面建成小康社会,率先迈向基本现代化,打造生态宜居型现代工业新城和国际化的文化旅游名城,建设富饶美丽幸福新湘潭。

　　主要预期目标:地区生产总值年均增长9%以上,三次产业结构调整为5:55:40;财政总收入年均增长9%左右;固定资产投资年均增长15%以上;社会消费品零售总额年均增长13%左右;进出口总额年均增长20%左右;城乡居民人均可支配收入年均分别增长9%和10%左右;户籍人口城镇化率提升到37%;万元GDP综合能耗五年下降17%,万元GDP二氧化碳排放五年下降18%,主要污染物排放完成省定任务;严守生态红线,森林覆盖率达到46.3%以上;城镇登记失业率控制在4.5%以内。

　　——打造智能制造谷。大力推进国家自主创新示范区建设,积极对接“一带一路”“中国制造2025”“互联网+”等战略,主攻智能制造,构建“1+4”特色工业体系,打造中部地区崛起智造谷。到2020年,智能装备制造产值达两千亿级,汽车及零部件、食品医药、新材料分别达到千亿级,新一代信息技术产值过500亿。布局完成“一轴一带”经济走廊。到2020年,“一轴一带”走廊工业总产值突破4000亿;湘潭高新区、湘潭经开区力争工业总产值分别达到1500亿左右,4个省级特色产业园工业总产值总计达到3000亿左右,其他各园区均成为地域核心增长极。实施工业强基和转型升级行动,培育发展机器人、3D打印、移动互联网、北斗导航应用、节能环保、新能源电池、高性能医疗器械等新兴产业,到2020年,高新技术产业增加值占比达36%以上。实施人才优先发展战略,坚持高瞻远瞩抓好高端人才的引进、培育和使用,打造人才高地,领军创新发展。

　　——打造精美新城市。围绕打造长江中游城市群核心引领区的目标,建设“精美城市”。总体布局“一核五城”,实施“一江两岸、北进中强,绿廊连接、组团发展”战略。以地标带动、功能提升、形象塑造、人文建设四大工程为抓手,推进海绵城市、绿色城市、智慧城市、文明城市建设,完成城市二环、湘江42公里风光带建设,实现建设精品化、设施精致化、管理精细化,成功创建全国文明城市、国家森林城市和国家历史文化名城。推进园区产城融合,实施“新城带动工程”,促进人口向新区聚集,使园区成为经济发展、人文活跃、生态宜居的新城区。做活湘江文章,深入实施湘江保护和治理“一号重点工程”,统筹全域生态建设和环境保护,完成“两型社会”建设改革目标任务。以长株潭共建百里滨江生态文化经济走廊为引领,加强湘江岸线规划建设,把湘江两岸打造成富有生态质感、景观魅力、文化内涵、现代气息的美丽舒适大气的诗意长廊。

　　——打造秀丽新农村。坚持统筹城乡发展,把“中国要强,农业必须强;中国要美,农村必须美;中国要富,农民必须富”的理念在湘潭落实好,补长全面小康建设的农村短板。促进城乡公共资源均衡配置,水、电、路、通信等基础设施加快向农村延伸,形成功能完善的城乡水利网、清洁安全的能源保障网、高速泛在的区域信息网。基本公共服务既要向农村全覆盖、提高供给总量,又要着力提高水平、实现质和量的城乡均等化。按照“规划引领、连片推进,产村一体、特色鲜明”的方略,开展美丽乡村建设。将美丽乡村与生态观光、休闲旅游、特色农业等结合起来发展,变“美丽”为生产力,建设一批经济强镇、经济强村,使农村既风光秀丽又有经济实力。到2020年,建成10个以上美丽乡村示范片,150个以上美丽乡村示范村,所有建制村基本完成人居环境整治任务,实现农村“生产美、生活美、生态美”。

　　——打造幸福新湘潭。坚持以人民为中心,共享发展,在经济繁荣、设施完善、环境优化、生态良好的基础上,着力提升群众生活水平,提前全面进入小康,率先迈向基本现代化。到2017年65个省定贫困村全部“摘帽”,10万贫困人口全部脱贫;到2020年居民生活总体富裕,城乡居民人均可支配收入分别达到4.5万元、2.5万元以上。公共服务更加健全,社会保障更加有力。高中阶段教育全面普及;城乡社会养老、医疗保险覆盖率达到100%,各项保障水平全省领先;中等偏下收入住房困难家庭住房保障实现应保尽保。促进人的全面发展,教育强市、文化湘潭、智慧湘潭、健康湘潭、平安湘潭等建设富有成效,社会温馨和谐、充满活力,全市人民生活更加幸福美满。

　　实现“十三五”蓝图,2016年开局十分关键。我们将认真贯彻落实中央、省委、市委经济工作会议精神,坚持稳中求进工作总基调,战略上坚持持久战,战术上打好歼灭战,着力加强结构性改革,提高供给体系质量和效率,提高投资有效性,着力培育新的发展动能,改造提升传统比较优势,增强持续增长动力。主要预期目标:地区生产总值增长9%;固定资产投资增长18%;规模工业增加值增长8.5%以上;财政总收入增长9%左右;社会消费品零售总额增长13%左右;进出口总额增长10%;城乡居民人均收入分别增长9%和10%。重点抓好以下工作:

　　(一)大力推进转型升级,在调结构中稳增长

　　建设国家自主创新示范区,打造“创新之都”。实质性推进国家自主创新示范区建设,厚植创新优势,建设创新型社会,着力将湘潭打造成“创新之都”。提升自主创新能力。以湘潭高新区为核心,协同全市创新资源,促进产业链、创新链、资金链融合发展。实施创新载体提升计划,新建一批国省级工程(技术)研究中心、重点实验室和重大科研设施。推进国家知识产权示范城市建设。积极培育和扶持以市场为导向的创新主体,鼓励引导企业持续开展研发活动,推进长株潭创新中心建设。全力支持驻潭高校健康发展,充分开发高校资源,促进高校与地方融合发展,推动产学研各方联合开展技术攻关,转化一批科技成果。以“高精尖缺”为需求导向,加强创新人才队伍建设。实施重大创新工程。围绕推动智能制造和传统产业转型升级,在集成电路设计、海工装备、车载传感系统和智能终端等领域突破一批关键技术。支持产业越界创新发展。推进市产业创新研究院、湘潭力合科技领航城建设,围绕新能源汽车、海泡石等领域组建专业研究所,加快实施一批产业化项目,创立海泡石工业园。推进军民融合,促进军民融合技术双向转移,重点推进工程机械设备、能源储运装备等关键技术和产品开发。优化创新创业环境。推动大众创业、万众创新。鼓励成立风险投资、创业投资等创新型金融机构,大力发展专利代理等中介服务机构,支持各类创新主体建设企业孵化器、创新创业苗圃、众创空间等服务平台,鼓励技术创新平台对外开放共享。孵化创新创业型企业1000家。在湘潭大学与湖南科技大学之间规划建设“创新创业街”,将之打造成湘潭“双创”新地标。建设中部地区崛起智造谷,推动产业向中高端迈进。大力发展智能制造产业。与上海交大等共建湖南智能制造创新中心。重点抓好海工装备、新能源装备、机器人等产业,加快高档数控机床、工业机器人等智能制造装备的研发与产业化,推进泰富重装二期、威胜电气产业园二期、吉利新能源SUV基地等项目建设。实施智能化改造提升工程。推动钢材及深加工、汽车及零部件等行业成套设备及生产系统智能化改造。将新一代信息技术贯穿于设计、生产、管理、服务等环节,加快智能车间和智能工厂建设。以“一谷四园”为主阵地,推进质量强市示范城市创建,提升产业整体质量水平。推动“制造+互联网+服务”融合创新,推进下一代互联网示范城市和“宽带中国”示范城市建设,加快“智慧湘潭”云计算数据中心和“无线湘潭”建设,开展互联网与工业融合创新试点,推动行业龙头企业开展物联网示范应用。促进企业创新发展。支持大型龙头企业通过合资、并购等方式整合市内外资源,实现低成本快速扩张。积极引导大型企业裂变发展,催生一批新型企业。推动迅达集团等一批科技型企业做大做强。健全中小企业融资服务体系,探索设立中小企业投资引导基金,引导大企业与中小企业开展协同合作。大力弘扬工匠精神,促进中小企业“专精特深”发展。全面落实国省惠企政策,开展降低实体经济企业成本行动,通过进一步清理规范中介服务、降低物流费用等途径,切实降低企业经营成本,为企业发展营造良好环境。

　　建设国家现代农业示范区,加快农业现代化进程。按照“一区三圈十园十片”现代农业空间布局,推进示范区建设。推动主导产业提质增效。突出发展设施农业、特色农业、观光休闲农业,重点抓好优质稻、蔬菜、湘莲、水果、茶叶、花卉苗木等一批现代农业产业示范片建设。推广优质稻180万亩,粮食总产稳定在150万吨左右。突出农业品牌培育和提升,加大沙子岭猪、湘莲等地理标志产品和龙牌酱油等“中华老字号”的开发力度。打造特色蔬菜93万亩,丰产油茶30万亩,丰产楠竹20万亩。加快转变农业发展方式。做好“接二连三”文章,鼓励和支持工商资本下乡,加快梅林桥、姜畲等10个现代农业综合示范园和加工物流园建设,支持伟鸿等农业产业化龙头企业发展,支持一批休闲农庄上档升级。扩大农机装备规模,重点解决生产关键环节的机械化问题,推进主要农作物生产全程机械化。推进土地整治、中低产田改造和高标准农田建设。开展重金属污染土地修复治理和种植结构调整试点,打造一批农业标准化生产基地。完善农业服务体系。支持华绿、雁芙等农业全程社会化服务组织、示范合作社加快发展。培养新型职业农民和农业职业经理人。加快电商直销、物流配送、期货涉农网购平台建设。支持农业自主创新,推进科技创新工程、农产品质量安全和品牌创建工程建设,增强农业现代化内生动力。

　　建设长株潭供应链核心枢纽,大力发展现代服务业。发展壮大现代商贸物流业。以打造长株潭供应链核心枢纽为龙头,培育壮大中国(中部)岳塘国际商贸城、义乌小商品城等专业市场,加快长株潭中央商贸区、湖南一力物流公路港、长株潭现代生态商贸新城等项目建设;改造提升建设路口、基建营等商圈;推进万达广场、湘潭中心等城市综合体建设,鼓励步步高、心连心等企业向多样化商业服务综合体转型。做大做强文化旅游业。以创建全国红色旅游融合发展示范区等为抓手,实施文化旅游产业重大项目带动工程,文化产业、旅游产业各引进投资20亿元以上项目1个或10亿元以上项目2个。突出韶山、昭山、隐山和水府庙水库、窑湾“三山一水一湾”旅游开发,抓好山市晴岚文化旅游小镇、文化艺术品交易中心、韶山黑石寨片区整体开发、水府旅游区和昭山设计创意产业园、媒体产业园等项目建设。推进盘龙大观园更好发展,支持盘龙大观园、齐白石艺术城联合窑湾文化旅游街区创建国家5A级旅游景区。以庆祝韶山灌渠通水50周年为契机,推进韶山银河旅游项目开发。积极培育高端服务业。重点发展科技与信息服务、电子商务、金融服务、会展、体育、健康养老等产业,抓好湘潭电商产业园、华拓万人级交付基地、湘潭金融商务区等项目建设,促进湖南大健康产业园、绿城养老、湘江国际会展中心等项目落地,支持星宇电商物流等电商企业发展,培育和强化现代服务业的核心增长能力。

　　(二)发挥“一轴一带”统筹作用,促进区域协调发展

　　园区协同发展。编制湘江高端服务创新产业轴和环线经济带规划,促进“轴带”产业布局。加强对沿轴连带园区的产业管控,统筹项目引进,强化招商合作和产业配套,力避内耗性竞争、产业雷同。大力实施“一区多园”,鼓励园区沿轴带发展“飞地经济”,共建共享。实施《园区发展三年行动计划》。湘潭高新区主抓新能源装备、精品钢材及深加工两个特色产业园建设,重点发展能源装备制造、新材料、现代服务业三大主导产业。湘潭经开区主抓海工装备、军民融合特色产业园建设,重点发展新一代信息技术、海工装备、节能与新能源汽车三大主导产业。昭山示范区重点发展生态文化旅游、文化创意、健康养生等现代服务业,打造绿色产业聚集区。其他园区按照各自产业定位,实现特色发展。推进创新创业园区发展“135”工程,年内新开工建设标准厂房50万m2以上,引进企业200家以上。

　　县域特色发展。充分发掘和整合县(市)优势资源,加快发展县域特色经济,逐步形成特色产业集群,促进县域崛起。湘潭县以天易示范区为中心,主动承接中心城区产业辐射,推进“一区多园”发展,抓好杨河、花石、青山桥等工业园建设,大力发展农产品加工型特色县域经济。湘乡市重点加快湘乡经开区建设和老工业区调整改造,培育壮大智能电气装备、生物医药、高端医疗器械等新兴产业,大力发展特色工业型县域经济。韶山市重点推进全域旅游,促进红色旅游、生态旅游、休闲旅游融合发展,不断延伸红色旅游产业链条,大力发展特色旅游型县域经济。

　　全域开放发展。积极对接“一带一路”、长江经济带发展战略,推进与沿边重点口岸、边境城市、经济合作区的合作。完善支持企业“走出去”的政策和服务保障体系,鼓励优势企业到境外上市、扩大海外投资和开拓国际市场。积极利用国际产能合作平台,以及省“装备产能出海行动”、“湘品出湘”工程等,推动企业和产品主动融入全球产业链、价值链、物流链,重点支持装备制造、生猪肉制品等高新技术及优势产品出口,推进服务外包示范基地建设,扩大服务贸易规模,加快外贸转型升级。提升湘潭综保区开放平台功能,抓好保税商品展示交易中心等项目建设,加快跨境电商、进口商品指定口岸、外贸物流平台的申报与建设,对接湘欧快线,完善与沿海口岸大通关协作机制,创新通关服务,提高贸易便利化水平。(三)努力扩大有效投资,增强持续发展动能突出重大项目建设。年内组织实施重点项目300个以上,力争完成投资900亿元以上。其中:实施重大产业项目150个左右,重大基础设施项目80个左右,生态环保和社会民生项目70个左右。加强政府投资项目计划管理,合理安排建设时序,避免临时动议。大力加强项目前期工作,采取市级领导专题协调、设立项目前期工作协调小组等方式,加强调度,提速项目手续办理,争取项目出库即可开工,全年安排重大前期项目200个左右,力争其中50个开工建设。强化市级领导联点主办制,推行“挂号销号制度”和“一线工作法”,全面落实集中服务、定期协调和限期办理等制度。加强开竣工管理。促进湘潭动力配件产业园、步步高全球采购仓储物流配送中心等100个项目开工建设,确保中国(中部)岳塘国际商贸城一期一阶段、屹丰汽车二期等50个左右项目竣工投产。

　　破解项目要素瓶颈。严格规范国土资源管理,积极争取用地指标支持,强化经营性用地集中统一管理,推进节约集约用地,依法加大对闲置低效用地的处置力度,确保重点项目用地需求。全力破解征拆难题,科学有序安排征收项目,严格执行全市统一的征拆政策,进一步强化征拆主体责任,明确进度节点,严格考核奖惩,加快拆迁腾地。加强项目建设资金保障。成立湘潭市重点建设项目股权投资基金,吸引更多社会资本参与公共项目建设。筹建湘潭市投融资服务中心,为项目单位和金融机构开展合作搭建新型平台。加大专项建设基金项目的申报力度,力争更多项目进入基金支持笼子。

　　强化战略招商引资。开展“招商引资突围年”活动,部门主动、全面招商。瞄准全球知名、行业领先的大企业,着力引进一批具有行业影响力、市场竞争力的大项目。国家级园区至少引进30亿元以上产业项目1个或20亿元以上产业项目2个;省级经济园区至少引进10亿元以上产业项目1个或5亿元以上产业项目2个。结合国省“十三五”规划,科学谋划和对接一批产业链上下游项目,特别是填补产业空白的重大项目,以及总部经济、研发中心等。加强与中建、中交、中航工业等央企战略合作,力争引进央企对接项目2～3个。创新招商方式,培育一批专业招商代理机构。健全项目引进、跟踪、促建、达产推进机制,提高招商履约率。强化招商统筹,建立项目定期发布和在谈项目申报制度,协同项目引进和布局。加强银企合作。推动险资入潭。力争全年引进市外资金300亿元以上。

　　(四)坚持以改革突破制约性问题,着力增强市场活力

　　深入推进经济体制改革。着力推进结构性改革,在供给侧和需求侧两端发力,逐步实现化解产能过剩、降低企业成本、消化库存、防范金融风险和补齐短板等目标。深化商事制度改革。探索建立市场准入负面清单管理模式,推进登记注册制度便利化。深化国企改革。积极推进新一轮国企国资战略重组;加快推进湘钢、湘电等驻潭国企分离办社会职能;推进混合所有制经济发展,鼓励社会资本以合资、参股等方式参与国企改革;以管资本为主加强国资国企监管。深化投融资体制改革。支持企业上市和新三板挂牌,积极拓展直接融资;

　　探索境外融资和融资性担保创新,支持城发集团等境外借壳上市、海外发债;规范PPP项目运作,在养老服务、教育信息化等领域务实推进。推进金融安全区和金融生态良好城市创建,加强全方位监管,规范各类融资行为,引导民间融资依法有序发展,积极防范金融风险。

　　深入推进财税管理体制改革。密切关注中央税制改革动向,进一步加大税收政策研究力度,逐步理顺税种增收管理关系。建立健全全口径政府预算管理体系,继续推进财政预决算信息公开,清理和规范同财政收支增幅或生产总值挂钩的重点支出,完善结转结余资金定期清理及预算编制挂钩机制,完善行政事业单位资产管理制度,提高财政资金效益。根据中央和地方事权与支出责任改革落地情况,合理确定市与县市区、园区之间的支出责任,逐步完善市对城区、园区的激励性转移支付制度。加强对园区财政收支及债务的管理。进一步推进和深化预算绩效管理,健全财政投资管理机制,制定出台政府非经营性投资项目代建制管理办法。完善政府债务管理机制,防范和化解政府性债务风险。

　　深入推进社会领域改革。规范有序开展政府向社会力量购买服务工作。深化教育教学改革,注重培养学生终身发展能力,全面提高教育教学质量,打造湘潭教育品牌。推进学区联盟和集团化办学。强化教育督导,深入推进管办评分离。优化城区高中学校布局。开展教师资格定期注册制度和人事工资分配制度改革。大力推进职业教育发展,优化职业院校专业设置,深化产教融合、校企合作,培育高素质劳动者和技术技能人才。以公立医院改革为重点,推进医疗、医保、医药“三医联动”改革,积极探索市级公立医院重组转型,深化新农合和医保支付制度改革。完成全市建制村区划调整。开展第三次全国农业普查。深化户籍和居住证制度改革,推进有能力在城镇稳定就业和生活的农业转移人口举家进城落户;实施以满足新市民住房需求、购租并举为方向的住房制度改革,加大棚改货币化安置力度,出台住宅验收综合管理办法,积极发展住房租赁市场,化解房地产库存。加快殡葬服务业改革。全面实施不动产统一登记制度。积极稳妥推进农民宅基地、林权和住房财产抵押、担保、转让,年内基本完成农村土地确权登记颁证工作。完善自然资源资产管理、排水许可、排污交易、生态补偿等机制。实行能源和水资源消耗、建设用地等总量和强度双控行动。基本完成绿色GDP改革试点。依法收回城区供水和污水处理特许经营权。

　　(五)抓好重大基础设施建设,优化发展空间格局强化规划引领。

　　完成城市总体规划修改。按照长株潭城市群发展要求,加强与长沙、株洲规划对接,促进一体化发展。推进宜居湘潭、特色湘潭规划,重点抓好棚户区和城中村改造规划、桥头环境综合整治规划、乡村规划等的编制,完成轨道交通、城市地下综合管廊等规划编制。保护和传承历史文化,建立城市历史人文与自然生态环境保护名录,加强传统村落民居和历史文化名村名镇保护。实行规划精细化管理,制定湘潭市实施《城乡规划法》细则、规划设计质量评价制度。强化规划执行刚性,加大执法力度,实行全过程、全覆盖的规划监管,确保规划严格落实。

　　完善交通网络。坚持长株潭一体化和市域五个县市区一体化同步推进,促进互联互通、内外畅通。长株潭城际铁路湘潭段年内通车。启动G60醴陵至娄底高速公路扩容工程湘潭段、S328韶山-月山-水府庙公路、武广高铁连接线、伏林大道等项目建设,加快推进益娄高速公路湘潭段、G320湘潭绕城线、湘乡至韶山城际快车道、长沙南横线至沪昆高铁韶山南站连接线、白云路、昭山北路、河口大桥、水府大桥、水府大道等项目建设,完成京港澳高速昭山北互通建设,S331湘乡-壶天公路一期、S332湘乡-棋梓桥等干线公路竣工通车。大力完善城市路网。启动文星门路、护潭西路、新马路等道路建设,推进湘江风光带、西二环一期、昭华大桥、杨梅洲大桥等“成环成带”道路和羊牯大道等城市出入口建设,熙春门北路、高新区滨江路等道路竣工通车。实施中心城区主次干道及重要节点提质改造“二年行动计划”。完成对芙蓉大道、湘潭大道、河东大道、双拥路等主次干道的提质改造及周边环境综合整治。全面启动一、二、三、四、五大桥匝道规划和建设。抓好湘潭易俗河港区一期、水上污染防治应急救援基地等水运项目建设。加快推进河东、河西综合客运站和沪昆高铁九华客运站、荷塘客运枢纽站场等建设。

　　提升承载功能。开展“城市建设提质年”活动,全力提高城市功能品质。把“两房两棚”“两供两治”作为重中之重,举全市之力,打造全省示范工程。抓好9个集中成片棚户区改造。抓好市第三水厂提质改造、河东第二污水处理厂、城区固体废弃物处置场所、生活垃圾无害化处理工程、天然气改扩建工程、“两供”管网改造工程等项目建设。推进综合管廊试点城市、海绵城市和公共停车场建设。大力推进综合运输服务示范城市建设,全面启动“713”示范创建项目,创新运输服务组织方式。优先发展公共交通,开通乡村公交线路,公交出行分担率提升到38%。推进城区公共自行车租赁系统建设。加强出租车管理,深入开展打非治违。加强城镇地下管线普查及市政公用设施安全监管。推进花石、棋梓、银田等特色重点镇建设,加大合并乡镇基础设施建设力度。加强农村基础设施建设。推进快递下乡工程。加紧建设农村公路会车点,建设农村客运招呼站300个。积极推进全国小农水重点县、全国城市水利规划试点市建设,建设水利工程3万处,抓好病险水库除险保安、农村渠系维护建设和农村饮水安全工程提质增效。推进全市生态河湖水系连通工程,启动韶山市城乡供水引调水工程建设。

　　建设美丽家园。全力推进全国文明城市创建,开展“创文关键年”活动。完成数字化城管体系建设。继续推进市容环境“五项”整治和道路超限超载、交通秩序等专项整治。对市域内高速公路两边房屋、城区湘江江边、城区公园周边环境等“三边”分期分批进行穿衣戴帽、提质改造和绿化美化。开展“拆违控违决胜年”活动,城区违法建设基本清零。坚持城市建设以自然为美,慎挖山、不填水,不栽“无头树”,多栽本地树种。推进宝塔公园、云盘公园、杨梅洲水上公园等建设,新建河滨公园、二桥北桥头公园,在羊牯塘片区规划建设生态游乐运动公园。建设城区西部近郊生态绿环。加快爱劳渠和百亩湖绿道、市二级渠道景观及泵站、王家晒撇洪渠和朝阳渠水系改造、碧泉湖景观改造等项目建设。推进争光渠、江麓两干渠、丰收渠、牟渠等黑臭水体整治改造。深入实施湘江保护和治理“一号重点工程”,推进竹埠港地区“退二进三”、锰矿地区综合整治、谭家山煤矿矿区综合治理等重大项目。全面完成湘江长沙综合枢纽工程库区污染综合防治。加强大气污染防治,空气质量优良率提高4个百分点。启动国家森林城市创建。推进韶山生态功能区建设。加快湘潭韶山植物园建设。加强昭山“绿心”保护。加强水府庙水库保护和综合治理。完成国省林业重点项目营造林8.2万亩。争取将仙女山风景名胜区申报为省级风景名胜区。执行“最严环保法”,落实生态环境损害责任终身追究制度和赔偿制度,由损害责任者承担赔偿责任,修复受损生态环境。推进梅林桥、姜畲、银田等美丽乡村示范片建设。深入开展农村环境卫生综合整治,开展清家园、清田园、清水源“三清”行动,持续改善乡村面貌。

　　(六)提升人民生活水平,凝聚全市发展合力

　　切实改善民生。推进“全国创业先进城市”和国省级创业孵化基地建设,落实创新创业“三年行动计划”,实现全年新增创业1万人以上。多渠道增加居民财产性收入,完善最低工资增长机制,促进居民收入稳步增长。落实城乡居民大病保险制度,推进机关事业单位养老保险制度改革,加快社保“一卡通”建设。提高城乡低保标准。深化“救急难”试点成果,推进重特大疾病医疗救助,实现医疗救助与基本医疗保险、大病医疗保险、商业保险有效衔接。推进住房公积金归集扩面,全面实施省内、跨省缴存异地互认和转移接续。强化仓储设施建设,完成新增地方粮食储备任务,确保农民粮食应收尽收。开展“扶贫攻坚年”活动,推进精准扶贫、精准脱贫,深入实施群众增收、异地搬迁、素质提升、兜底保障、基础扶贫等“五大工程”,健全金融服务、社会参与、责任考核、建档立卡、贫困对象退出等精准扶贫机制。

　　继续办好十件民生实事:新增城镇就业5万人,扶持返乡农民工创业基地10个,新增农村劳动力转移就业1万人;建设义务教育合格学校23所、农村公办幼儿园7所;新开工各类保障性住房和棚户区改造10000户,农村危房改造2000户,资助农村特困户建房200户;对城区16处交通拥堵路段和节点实行疏堵保畅措施,创建公交车、出租车为民优质服务示范岗位200个;城乡低保、农村五保户保障标准提标,贫困残疾人家庭无障碍改造300户;农村危桥改造29座;新建城乡广场文化和体育健身设施各30处,建设社区矫正中心4个,建设便民服务示范社区4个;精准扶贫实现贫困人口脱贫30631人;为全市60周岁以上失独老人提供扶助,为城乡低保困难家庭孕妇免费提供产前筛查,建立贫困危重孕产妇救助机制,开展孕产妇孕产期健康知识普及行动;推进村级组织活动场所和乡镇机关“五小设施”提质改造,调剂、划拨、新(改、扩)建一批社区公共服务场所。

　　提升公共服务水平。促进教育公平,推进城乡教育优质均衡发展,推行义务教育学校校长、教师交流轮岗制度。新(改)建风车坪建元学校等7所学校,城区新增义务教育学位2390个。采取政府购买服务方式解决城乡中小学生“午托难”问题。深入开展健康教育,推进健康城市建设。开展“进一步改善医疗服务行动”,提高医疗服务质量;大力发展中医药事业,提升创建全国基层中医药工作先进市成果;加快市一医院全科医生临床培养基地、市公共卫生服务中心等项目建设。彻底解决市中心医院进出通道拥堵问题。推进医卫信息平台建设,促进远程医疗服务。全面落实大病医疗保险和医疗责任保险。鼓励社会力量兴办健康服务业。推进医养融合,建成第六人民医院康复大楼,完成市福利院老年养护楼后续工程建设。实施“全面二孩”政策,整合卫生计生优质服务资源,提高人口素质,促进人口均衡发展。健全农村留守儿童、妇女、老人关爱服务体系和扶残助残体系。深化全民健身示范城市建设,大力开展“欢乐潇湘·幸福湘潭”“全民健身日”等文体活动,办好市十三运会。倡导全民阅读,建设书香社会。加快档案馆新馆建设,启动图书馆新馆、科技馆、工人文化宫建设。加快构建覆盖城乡、惠及全民的公共法律服务体系,满足人民群众基本法律服务需求。

　　全面加强和创新社会治理。在党委统一领导下,大力提高社会治理法治化水平。创新基层社会治理模式,健全“三社联动”工作机制,加强社区公共服务综合信息平台建设。规范居民小区物业管理。加快公安民警训练基地、强制隔离戒毒所二期建设,建好城区派出所8个、社区警务室79个。规范社会组织管理。全面深化社会诚信体系建设,弘扬商业诚信。加强市场综合监管。保护消费者合法权益,建立社会共治的维权机制。提高食品药品科学监管能力,推进食品安全追溯系统建设,全面保障饮食用药安全。完善危机预防和管理体系,提高公共安全等预防和应急处置能力。建立风险识别和预警机制,提高风险防控能力。启动“七五”普法,持续提高全民法治素质。深入推进信访信息化建设,强化诉访分离,引导群众依法逐级信访。实施最严格的安全生产管理责任制度,实行党政同责、一岗双责、失职追责,强化依法治理,铁腕推进打非治违和专项整治,堵塞各类安全漏洞,确保安全形势持续稳定好转。推进平安湘潭建设,完善立体化社会治安防控体系,实行网格化管理,深入开展“三年禁毒大行动”,严密防范、依法打击各类违法犯罪活动,切实维护社会和谐稳定。

　　加强国防动员、民兵预备役和人防工作,推动军民融合深度发展。广泛开展全民国防教育和“双拥”共建活动,密切军政军民团结,更好发挥军队在支持地方经济社会发展中的作用。三、加强法治政府和服务型政府建设

　　为了圆满完成“十三五”规划和2016年各项目标任务,必须坚持法治、服务、效能等原则,建设一个充满活力、富有效率,能够带领全市人民创新创业、实现率先发展的政府。

　　以法治建设为根本,完善体制机制。坚持在党的领导下、在法治轨道上开展工作。全面贯彻依法治国方略,加快法治湘潭进程,建设权责法定、执法严明、守法诚信、公开公正的法治政府。以法治为根本遵循,建立健全决策、执行、公开、考核等工作机制,坚决做到决策时依法、作为时用法、全过程守法。依照法定原则,加快部门职能调整,严格职权配置,健全规章制度,推进机构、职能、权限、程序、责任法定化,切实做到“法定职责必须为、法无授权不可为”。完善权力监督机制。与检察机关共同推进行政执法与刑事司法信息共享平台建设。主动接受人大、政协和社会各界监督,加强审计工作,加大行政监察力度,形成科学有效的权力运行制约和监督体系。严格考核问责,实行更加科学的绩效考核制度,健全重大决策部署执行情况检查和专项督查制度,促进政府规范、高效、透明用权。

　　以增强活力为目标,优化政府管理。坚持简政放权、放管结合、优化服务,使政府的手成为市场活力迸发的推手。大力推行“清单”管理,精简行政审批,规范中介服务,放开市场主体的手脚,做到“法无禁止即可入”,用政府权力的“减法”换取市场活力的“乘法”。加强政府诚信建设。爱护创业者,尊重企业家,保护合法财产。防止对企业的不当干预。促进市场主体守法经营。推进公共资源市场化配置,依托公共资源交易中心,使每一笔公共资源交易都规范化、公开化。把政府该管的事管好,健全监管制度,保护合法,打击非法,使市场既开放竞争,又运转有序。牢固树立“以相对人为中心”的行政服务理念,优化服务流程,加强市级政务数据中心和各级各部门政务网站集约化建设,实现服务的人性化、简约化、标准化、制度化。

　　以率先发展为追求,加强能力建设。提前全面建成小康、率先迈向基本现代化,必须有过硬的能力作保障。要提高谋划发展的能力,善于抓住关键问题,立足全局,善谋善断,增强发展的前瞻性、整体性和系统性;要提高抢抓机遇的能力,善于在国际国内大势中捕捉机遇,把优势产业“抢”到湘潭来,把重大项目“抢”到湘潭来,把政策资源“抢”到湘潭来;要提高创新创造的能力,既讲规矩,又善于结合实际出点子、谋突破,以重点突破带动全面发展;要提高攻坚克难的能力,培养“狮子型”干部作风,能担当,敢碰硬,没有拿不下的堡垒;要提高抓落实的能力,对上级决策部署,令行禁止,雷厉风行,用实绩来回话。关心基层干部,加强基层队伍建设,提高基层搞服务和抓发展的能力。通过强化能力建设,为率先发展提供坚强的保障。

　　以清廉政府为标杆,引领社会风尚。坚持改革各项举措都体现惩治和预防腐败的要求,同步考虑、同步部署、同步检查,堵塞一切可能出现的腐败漏洞,让治标与治本结合,改革与廉洁同行,把清廉政府建设作为标杆在全市人民面前树起来。坚决做到干部清正。政府领导率先垂范,以更高更严的要求,履行“两个责任”,自觉把纪律和规矩挺在前面;全体干部职工要认真学习遵守法律法规和制度,依法依纪行权履职。坚决做到政府清廉。巩固党的群众路线教育实践活动成果,坚决反对“四风”,厉行勤俭节约,力戒奢侈浪费,从严控制行政支出,挤出更多的财力用于民生社会建设;全面加强惩治和预防腐败体系建设,强化正风肃纪,坚决查处违法违规行为。坚决做到政治清明。深入落实“三严三实”要求,坚持为民务实,整治机关“庸懒散拖”等不良作风,既要反腐败,又要防溃败;要恪守为民之责,做人讲良心,做官讲民心,政府的心始终要和老百姓的心贴在一起,使“清风”能够吹进“民风”,使干部和群众真正形成“鱼水情”,形成共建美好家园的强大合力和良好风尚。

　　各位代表,“十三五”发展蓝图已经描绘,美好的前景催人奋进。我们满怀信心迈上了新的征程。让我们更加紧密地团结在以习近平同志为总书记的党中央周围,在中共湘潭市委的坚强领导下,万众一心,开拓前进,为提前全面建成小康社会、率先迈向基本现代化而奋斗!
